# Complete mitochondrial genome of the earthworm *Amynthas seungpanensis* (Clitellata: Megascolecidae)

**DOI:** 10.1080/23802359.2022.2080604

**Published:** 2022-06-10

**Authors:** Min Jee Kim, Yong Hong

**Affiliations:** a Experiment and Analysis Division, Honam Regional Office, Animal and Plant Quarantine Agency, Gunsan, Republic of Korea; b Department of Agricultural Biology, College of Agriculture & Life Sciences, Jeonbuk National University, Jeonju, Republic of Korea

**Keywords:** *Amynthas seungpanensis*, mitochondrial genome, Megascolecidae, phylogeny

## Abstract

*Amynthas seungpanensis* Song and Paik, 1970 (Clitellata: Megascolecidae) is an endemic and ecologically important species in Korea; however, knowledge regarding its genetic characteristics is lacking. In this study, we sequenced the complete mitochondrial genome (mitogenome) of *A. seungpanensis*. The mitogenome is 15,085-bp long and contains 13 protein-coding genes (PCGs), 2 rRNA genes, 22 tRNA genes, and 1 major non-coding region, the control region. This mitogenome has an arrangement identical to that observed in the mitogenomes of most earthworms, and all 37 genes are transcribed from the same directional strand. All 13 PCGs have the same start codon (ATG). Six PCGs (*COI*, *COII*, *ND6*, *CytB*, *ATP6*, and *ND4*) end with TAA and *ND4* ends with TAG, whereas the remaining genes exhibit an incomplete stop codon, T. The A/T content of the complete mitogenome is 65.8%; however, it varies among regions and genes as follows: control region, 77.8%; srRNA genes, 65.8%; lrRNA genes, 68.8%; tRNA genes, 64.2%; and PCGs, 65.0%. Results of phylogenetic analyses suggested the family Megascolecidae to be a monophyletic group with the highest nodal support, whereas the genus *Amynthas*, which belongs to Megascolecidae, was determined to be non-monophyletic.

*Amynthas seungpanensis*, which belongs to the earthworm family Megascolecidae, is an endemic species in South Korea and was first discovered in Seongpanak, Mt. Halla. Mt. Halla is a dormant volcanic mountain on Jeju Island, South Korea, with the highest peak at 1950 m above sea level. This species is spotted rarely and is mainly observed during from the month of July to early September in the forests of Mt. Halla, South Korea (Song and Paik 1970). Although it is difficult to spot *Amynthas* adults, they can usually be observed at a specific time, especially after a heavy rain in late August. The body length of the species ranges from 105 to 120 mm, width from 5 mm to 6 mm, and segment length from 78 to 98 mm (Song and Paik, 1970). Earthworm groups in the forests of South Korea are dominated by the species of the genus *Amynthas*. This group is diverse and abundant in the litter layers and soil of the forests. Morphologically, the shape of the male pore, especially the male disks, is useful for the taxonomical characterization of the genus *Amynthas* and has been used throughout history of the identification of the species of this taxon. The male disc-shaped *A. seungpanensis* is unique and can be easily distinguished from other endemic Korean earthworm species. Currently, the complete mitochondrial genome (mitogenome) sequences are available for only 20 species of Megascolecidae (Boore and Brown 1995; Wang et al. 2015; Zhang et al. 2016a, 2016b; Unpublished, GenBank numbers KP688581, KP688582, EF494507). Accordingly, the characterization of the mitogenome sequences of more species of Megascolecidae is required for potential use in mitogenome-based phylogeny and for further understanding Clitellata mitogenomic evolution.

One *A. seungpanensis* adult was collected at Mt. Halla, Jeju-do Island, Korea (33°2250.82″ N, 126°3718.70″ E; 704 asl) on 30 August 2019. A voucher specimen was deposited at the Jeonbuk National University, Jeonju City, Korea, with the accession number JBNU0003 (Yong Hong, yonghong@jbnu.ac.kr). This species was collected from the mountains of Korea; there were no restrictions for species collection. Mitogenome sequences were generated using the MGISEQ2000 system with 150-bp paired-end reads and assembled by *de novo* assembly using a GenBank-registered earthworm mitogenome. Phylogenetic analyses were performed using 21 mitogenome sequences of species from the Megascolecidae family, including *A. seungpanensis.* Whole mitogenome sequences were aligned, and unaligned regions were removed using Gblocks (13,897 bp, including gaps). The Bayesian inference (BI) method, implemented in CIPRES Portal v. 3.1 (Miller et al. 2010), was used for phylogenetic analyses with a substitution model (GTR + Gamma + I).

The 15,085-bp long complete mitogenome of *A. seungpanensis* (GenBank accession number OL321943) consisted of 2 rRNAs, 22 tRNAs, 13 protein-coding genes (PCGs), and one major non-coding control region. The arrangement of the *A. seungpanensis* mitogenome and other available mitogenomes of the members of Megascolecidae showed an identical gene arrangement; all 37 genes are transcribed from the same strand (Boore and Brown 1995; Wang et al. 2015; Zhang et al. 2016a, 2014b; Unpublished, GenBank numbers KP688581, KP688582, EF494507). The size of the genome is well within the range found in species from Megascolecidae, which ranges from 15,013 bp (*Amynthas robustus*; GenBank Acc. no. KT429019) to 15,188 bp (*Amynthas pectiniferus*; GenBank Acc. no. KT429018). All 13 PCGs started with ATG, which is typical in invertebrate mitochondrial PCGs. Only six PCGs ended with the complete stop codon TAA and ND4 ends with TAG, whereas the remaining ones have an incomplete stop codon, T. The A/T content of the whole mitogenome was 65.8%, which is well within the range found in Megascolecidae species (63.0–66.3%; Zhang et al. 2016a, 2016b) and varied among the region/genes as follows: control region, 77.8%; *srRNA*, 65.8%; *lrRNA*, 68.8%; tRNAs, 64.2%; and PCGs, 65.0%.

Phylogenetic analyses placed *A. seungpanensis* in Megascolecidae as a sister group of *A. cucullatus*, *A. carnosus*, *A. cortices*, *A. longisiphonus*, and *Duplodicodrilus schmardae*, with this relationship demonstrating a high nodal support in BI analyses (Figure 1). Additionally, the family Megascolecidae represented a monophyletic group with the highest nodal support (1 in Bayesian posterior probability), whereas the genus *Amynthas*, which belongs to Megascolecidae, represented a non-monophyletic group.

**Figure 1.**
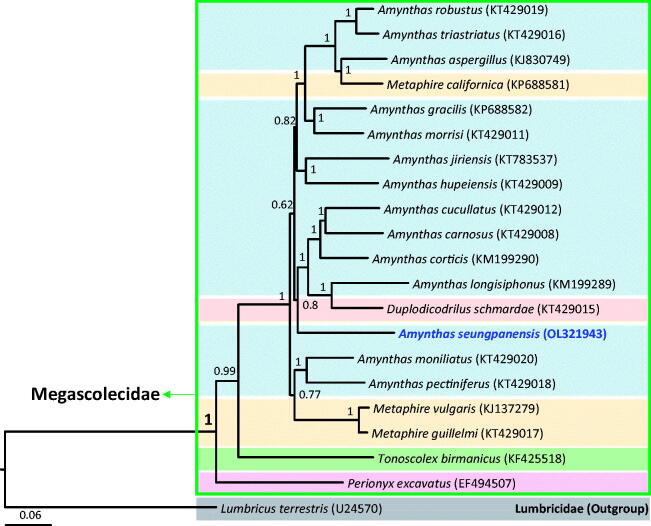
Phylogenetic tree of Megascolecidae. Bayesian inference (BI) method was used for the phylogenetic analysis based on whole mitogenome sequences. The numbers at the nodes indicate Bayesian posterior probabilities by BI. The scale bar indicates the number of substitutions per site. Each color bar indicates a specific genus. Lumbricidae (*Lumbricus terrestris,* U24570, Boore and Brown 1995) was used as the outgroup.

## Author contributions

Conceptualization, M.J.K. and Y.H.; methodology, M.J.K. and Y.H.; data analysis, M.J.K.; investigation, M.J.K.; resources, Y.H.; data curation, M.J.K.; − original draft preparation, M.J.K.; − review and editing, Y.H.; project administration, Y.H.; funding acquisition, Y.H. All authors have read and agreed to the published version of the manuscript.

## Data Availability

The data that support the findings of this study are available at the US National Center for Biotechnology Information (NCBI) database at https://www.ncbi.nlm.nih.gov/nuccore/OL321943. The GenBank accession No. (reference number) was OL321943. The associated BioProject, Bio-Sample, and SRA numbers are PRJNA796048, SAMN24731135, and SRR17621386, respectively.
